# Photoelectrochemical Production of Peroxydisulfate (PDS), a Clean Oxidant: Recent Development and Challenges

**DOI:** 10.3390/ijms27073066

**Published:** 2026-03-27

**Authors:** Zeeshan Haider, Muhammad Imran, Tahir Muhmood

**Affiliations:** 1 Department of Physics and Semiconductor Science, Gachon University, Seongnam-si 13120, Republic of Korea; 2 Gachon Bionano Research Institute, Gachon University, Seongnam-si 13120, Republic of Korea; 3 Department of Chemistry, Faculty of Science, Research Center for Advanced Materials Science (RCAMS), King Khalid University, P.O. Box 960, Abha 61421, Saudi Arabia; 4 International Iberian Nanotechnology Laboratory, 4715-310 Braga, Portugal

**Keywords:** peroxydisulfate (PDS), photoelectrochemistry, anion oxidation, sustainable oxidant synthesis, WO_3_

## Abstract

Peroxydisulfate (PDS, S_2_O_8_^2−^) is an important oxidant for a wide range of industrial applications, including organic synthesis, polymer preparation, wastewater treatment and environmental remediation. Currently, PDS is commercially produced by electrolysis of sulfate solution. Photoelectrochemistry (PEC) provides an alternative approach to PDS generation by reducing the energy required to drive this process. Because PEC uses solar light as an abundant, free resource, it is an attractive technique for PDS generation compared to electrolysis. WO_3_, owing to its excellent stability in acidic environments, is an excellent metal oxide candidate for producing PDS. Withstanding stronger acidic pH as well as absorption of visible light as a major fraction of solar light renders WO_3_ a promising material for PEC-based PDS production when compared with other semiconductors. This mini review examines light-assisted, sustainable production of PDS on WO_3_ photoanodes. It mainly involves the oxidation of the anion bisulfate, HSO^4−^, in a highly acidic pH. The efficiency of photoelectrochemical generation of PDS is greatly influenced by important factors, including suppressing recombination of photoinduced charge carriers, cocatalyst loading, minimizing competing side reactions, and establishing coupled reactions. In this review, we briefly discussed the key highlights to date in the application of WO_3_ as a stable photoanode material for producing PDS. It provides insight into the potential of photocatalysis as an emerging route for the sustainable synthesis of PDS as a valuable chemical oxidant. Besides the significant progress made so far, the PDS production rate remains low, and minimizing the recombination tendency to achieve a higher photocurrent density could further boost PEC-based PDS production.

## 1. Introduction

### 1.1. Importance of Peroxydisulfate (PDS) and Its Applications

Peroxydisulfate (PDS), a dimeric peroxide of sulfate and a strong oxidant, is commonly used as the sodium [1], potassium [2], or ammonium salt [3]. Peroxydisulfate is sometimes also referred to simply as persulfate [4]. The standard reduction potential of the S_2_O_8_^2−^ anion (2.1 V) is higher compared to that of H_2_O_2_ (1.8 V) [5]. It enables PDS to be used as a potential oxidant for diverse applications. The ·OH radical-mediated oxidative degradation of organics is an important route for decontamination [6]. PDS is commonly applied in sulfate radical-mediated degradation of organics [7]. Sulfate radicals have a high oxidation potential compared to hydroxyl radicals [8]. It can be activated through electron or energy transfer processes to produce sulfate radicals, which can effectively degrade organic contaminants [9]. PDS is frequently applied for the degradation of dyes and organic pollutants [10]. Besides AOP, PDS is also commonly applied as an oxidative reagent in sulfate radical-mediated organic synthesis [11,12,13], heterocycle formation [14] and as an initiator in polymerization [15] and hydrogel formation [16].

### 1.2. Limitations of Conventional Approaches for PDS Production

**Figure 1:**
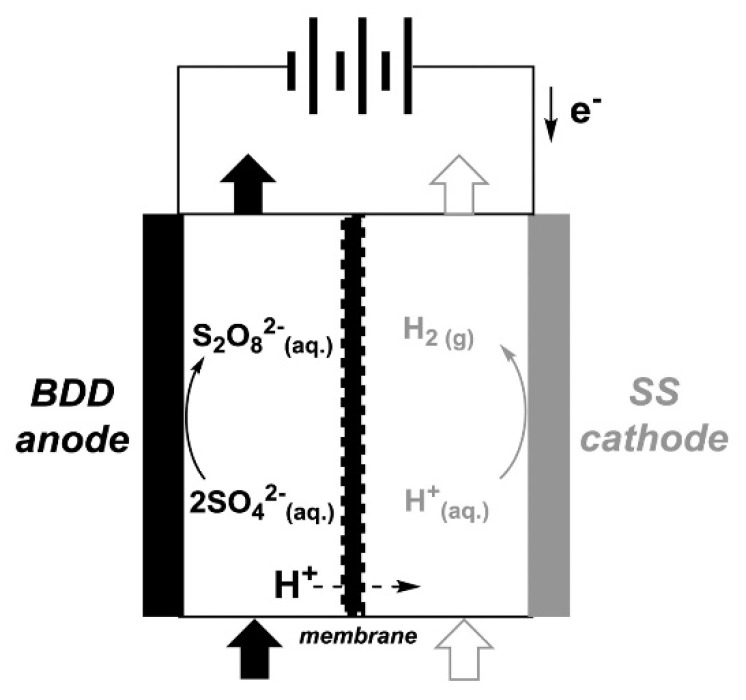
A representative electrochemical cell for the production of peroxydisulfate, adopted from reference [17].

Traditionally, PDS is produced through electrochemical methods [17,18,19,20]. It involves anodic oxidation of sulfate (SO_4_^2−^) and hydrogen sulfate or bisulfate (HSO_4_^−^) anions, as shown in the following equations [21]. A typical electrochemical cell applied for the electrochemical production of PDS is shown in Figure 1.

$$2\;{\mathrm{SO}}_{4}^{2-}\to S_{2}O_{8}^{2-},\;2\;e^{-},\;E^{o}=2.01\;V$$

$${\mathrm{HSO}}_{4}^{-}\to S_{2}O_{8}^{2-}+2H^{+}+2e^{-}\;E^{o}=2.12\;V$$

Various electrode materials have been used to produce PDS to date. This includes Pt [22], Ti/IrO_2_ [23], Ti/RuO_2_ [24], boron-doped diamond (BDD) [25] and TiO_2_ [26].

There are certain limitations in the electrochemical synthesis of PDS, including [27] (i) high amount of overpotential needed to drive this reaction, (ii) high cost of conventionally adopted electrodes, (iii) competition with oxygen evolution reaction (OER) at anode (iv) decreasing current efficiency over the elongated electrolysis for PDS production and (v) requiring higher in-put of electrical energy.

### 1.3. PEC Anion Oxidation vs. Water Oxidation

**Figure 2:**
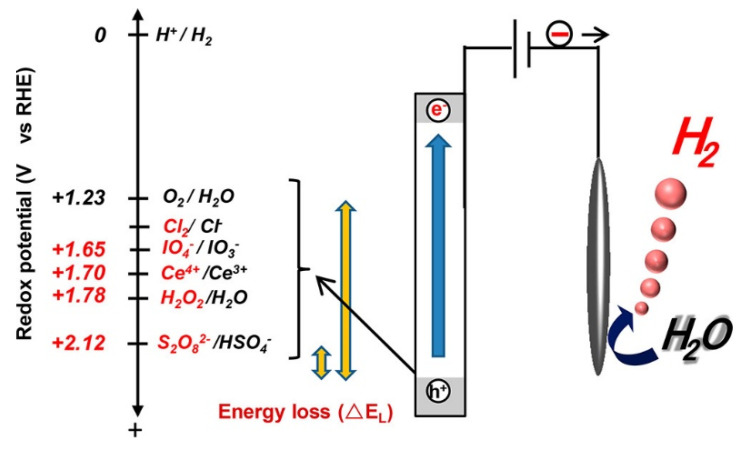
Principles of producing high-value-added chemicals on photoanodes adopted from reference [36].

The use of light to catalyze chemical conversions is a very sustainable approach for chemical synthesis [28]. Photoelectrochemistry (PEC) is a very useful strategy to produce PDS based on exploiting earth-abundant metal oxides [29]. PEC strategy can be considered as a hybrid approach that utilizes both electrical energy and a light source [30,31]. It reduces the amount of input electrical energy, making it economically feasible [32]. Metal oxides have been extensively applied as photoanodes for PEC water oxidation to produce oxygen via the oxygen evolution reaction (OER) [31,33,34]. However, at the photoanode, by replacing OER, value-added chemicals can be produced, as shown in Figure 2. Among these products, PDS is especially important given its wide range of applications. Producing PDS instead of oxygen at the photoanode is more economically valuable. PEC-based PDS production at oxide photoanodes may face the OER as a competing side reaction. PDS production and OER both are derived from photogenerated holes. The oxidation potential of S_2_O_8_^2−^/HSO_4_^−^ and O_2_/H_2_O are +2.12 and +1.23 V respectively vs. RHE. It indicates that PDS production occurs at a higher potential than OER, and the former reaction is thermodynamically less favored [35,36]. Therefore, careful attention to tuning reaction conditions and modifying the anode material is needed to maintain higher selectivity for PDS production and to reduce the oxygen evolution from water splitting as a competing side reaction. Although photoelectrochemistry at oxide photoanodes has been extensively investigated for oxidation of water to produce oxygen gas as a product, the application of these electrodes for alternate oxidation reactions, such as PDS generation, has been relatively less explored [37,38,39]. However, producing PDS rather than oxygen at the photoanode is of more economical value [40,41]. Therefore, emphasis in this direction could open new opportunities to apply photoelectrochemistry for sustainable catalysis.

### 1.4. Acidic Stability and Band Alignment of WO_3_ for PEC-Based PDS Production

Although several oxide photoanodes have the potential to be exploited for PEC-based PDS generation, there are certain conditions that should be met for the consideration of an ideal candidate to drive this reaction. Many of the commonly applied photoanodes, such as ZnO [42,43], Fe_2_O_3_ [44] and BiVO_4_ [45,46] for PEC water splitting, cannot withstand the stronger acidic pH needed for PDS production. For instance, ZnO is considered a stable oxide for solar cells [47]. It is also commonly used for solution-phase photochemical reactions such as photodegradation of dyes [48]. However, it can be corroded under stronger acidic electrolytes [49], limiting its use in wide pH ranges. Therefore, such oxide semiconductors may not be suitable for driving PEC-based PDS production, unless their stability is enhanced through realistic modifications under acidic electrolyte conditions [46]. Furthermore, the photoanode material should have sufficient thermodynamic potential to drive the formation of PDS. Titanium dioxide (TiO_2_) also has relatively higher stability in acidic electrolytes [50]. However, the drawback associated with TiO_2_ is its wide band gap (3.2 eV) and absorption of ultraviolet radiation, which is a smaller fraction of solar radiation (<3%) [51]. Tungsten trioxide (WO_3_), due to its narrow band gap (2.5–2.8 eV), can absorb visible light [52], which accounts for about 43% proportion of the solar spectrum [53]. WO_3_ as an important oxide is commonly applied in optoelectronics and electrochromic devices [54,55]. Considering solution phase chemistry, WO_3_ exhibits relatively higher stability under acidic conditions [56]. Moreover, its thermodynamic potential is also sufficient to drive PEC-based PDS production [57]. It makes WO_3_ an attractive condition to produce PDS through a photoelectrochemical approach.

In this review, we have briefly discussed the advancements made so far in driving photocatalytic PDS production over WO_3_-based materials. It includes the photochemical and photoelectrochemical approaches for producing PDS over the WO_3_ photocatalyst. We have discussed the mechanism involving PDS production over WO_3_ photocatalysts. Various strategies and approaches, such as co-catalyst loading, work functional tuning, blocking layer to prevent charge recombination, coupled photo redox reactions and fabricating three-dimensional hierarchical structure, have been discussed. We have also discussed existing limitations and proposed new strategies to make significant advancements in this research direction.

## 2. Fundamental Mechanism of PDS Formation on WO_3_

WO_3_ photocatalyst upon excitation generates holes in the valence band and electrons in the conduction band. As shown in Equation (1), the amount of potential required for converting HSO_4_^−^ anion to S_2_O_8_^2−^ is 2.12 V, whereas the valence band potential of WO_3_ is much more positive (ca 2.97 V vs. NHE at PH = 0), which is required to drive this reaction [35]. Hence, photogenerated holes in VB of conduction band drive the oxidation of HSO_4_^−^ anion to S_2_O_8_^2−^. Since photogenerated holes and electrons have a tendency to recombine with each other, if the conduction band electrons are utilized for coupled reactions such as oxygen reduction reaction (ORR) facilitated by a co-catalyst such as Pt, it can discourage the recombination of electron and hole pair, which can promote the formation of PDS. However, for the case of photoanode-driven PDS production, application of external bias facilitates the separation of charge carriers.

## 3. Advancements in Dispersed Powder Photocatalytic Systems

Loading metal-based co-catalysts is a promising approach to suppress the recombination of electron–hole pairs, thereby promoting the oxidative capability of WO_3_ photocatalysts [58]. An overview of WO_3_-based photocatalysts for producing PDS under illumination is summarized in Table 1. 

**Figure 3:**
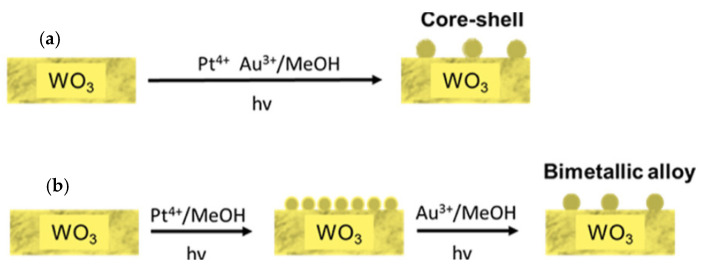
Fabrication Process of (**a**) Au@Pt/WO_3_ and (**b**) Pt–Au/WO_3_ adopted from reference [59].

**Figure 4:**
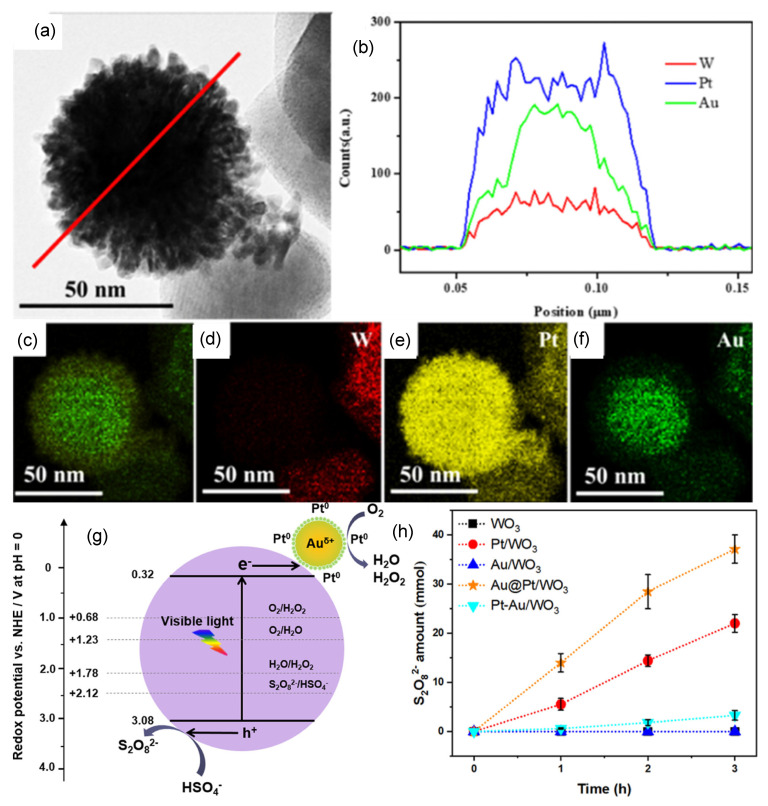
(**a**) selected TEM image for mapping; (**b**) line scan; (**c**) EDS combination of Pt and Au elements; (**d**) W element; (**e**) Pt element; and (**f**) Au element distribution of Au@Pt/WO_3_. (**g**) proposed photocatalytic generation of S_2_O_8_^2−^ and H_2_O_2_ over Au@Pt/WO_3_ and (**h**) time course of S_2_O_8_^2−^ amount under 3 h of irradiation adopted from reference [59].

Huang et al. have demonstrated the effectiveness of loading bimetallic Au and Pt nanoparticles over WO_3_ (Au@Pt WO_3_) for PDS generation in a powdered photocatalytic system [59]. In this bimetallic design, Au served as the core and Pt as the shell. It has been helpful to modulate the electronic states of Pt and expose the active catalytic sites by efficient collection of photogenerated electrons. The scheme for the fabrication of Au@Pt/WO_3_ and Pt-Au/WO_3_ is shown in Figure 3. TEM image, line scanning and elemental mapping of Au@Pt/WO_3_ are shown in Figure 4a–f. It was observed that Au existed mainly in the core, and Pt was uniformly distributed. Loading of such core–shell nanoparticles over WO_3_ has resulted in a significant improvement in photocatalytic PDS production, which was found to be 1.7 times higher as compared to Pt-loaded WO_3_ (Pt/WO_3_). It has produced a quantum efficiency of 5.2% at 420 nm. It is notable that the performance of Au@Pt WO_3_ for PDS generation was superior to that of introducing Pt-Au bimetallic alloy over WO_3_. In this setup, electrons were involved in the oxygen reduction reaction over Au@Pt, and photogenerated holes actively participated in the oxidation of HSO_4_^−^ anion to S_2_O_8_^2−^ species. Effective electron trapping is essential for avoiding the recombination with holes, which can support higher oxidation of HSO_4_^−^ anions. The mechanism involving the photoredox reaction over Au@Pt WO_3_ for PDS generation is shown in Figure 4g. Time-dependent production of PDS over various samples given in Figure 4h shows that the highest activity was observed for the Au@Pt WO_3_, which was found to be much higher than all other samples, including WO_3_, Pt/WO_3_, Au/WO_3_, as well as Pt-Au/WO_3_. Even though Pt-Au/WO_3_ contains both Pt and Au, a negligible amount of PDS generation over it reflects that rational structure design of bimetallic Au and Pt structure is important for optimizing the photocatalytic activity of WO_3_ towards PDS generation. Although Pt is an important co-catalyst for trapping the electron, it is important to control the form of Pt existing on the catalyst to precisely control the selectivity of photo redox reactions. The higher activity of Au@Pt/WO_3_ was due to the formation and exposure of higher Pt0 at the surface. 

Huang et al. have further demonstrated powder-based photocatalyst systems for PDs generation over WO_3_ modified with noble metals [60]. For that purpose, a photo deposition strategy was adopted to introduce Pt, Au, Ru, and Rh over WO_3_. For the sake of comparison, H_2_WO_4_, BiVO_4_, g-C_3_N_4,_ and P-25 were also tested for their activity toward PDS generation. A photocatalytic test was performed by dispensing catalysts in H_2_SO_4_ or NaHSO_4_ aqueous solutions, and O_2_ was purged during reaction to drive the ORR to utilize photogenerated electrons and avoid their recombination with photogenerated holes. The catalyst efficiency was analyzed by measuring S_2_O_8_^2−^ concentration and determining the quantum efficiency at 420 nm. The stability of the photocatalyst was examined by repeating the 6 cycles and analyzing the XPS spectrum before and after photocatalysis to determine the surface state of the photocatalyst. Photo deposition has resulted in the deposition of Pt nanoparticles tens of nanometers in size on WO_3_ particles. Under O_2_ purging conditions, bare WO_3_ has produced a negligible amount of S_2_O_8_^2−^, whereas Pt-loaded WO_3_ has shown significant improvement in performance, resulting in a yield of 33.9 µmol of S_2_O_8_^2−^ during 3 h of photocatalytic reaction in the presence of H_2_SO_4_. The corresponding quantum efficiency was observed to be 3.8%. It is notable that a quite similar yield (33.0 µmol) of S_2_O_8_^2−^ was observed when NaHSO_4_ was used instead of H_2_SO_4,_ reflecting that S_2_O_8_^2−^ production is mainly dependent upon HSO_4_^−^ anions. Compared to Pt, other noble metals have shown very low performance. Similarly, other photocatalysts such as TiO_2_, BiVO_4_, and g-C_3_N_4_ have shown much lower performance than WO_3_.

It confirms WO_3_ and Pt are appropriate catalyst and co-catalyst, respectively, for producing S_2_O_8_^2−^. Another important aspect authors have demonstrated is the capability of noble metal-loaded WO_3_ to drive oxygen reduction reaction (ORR), to consume the photogenerated electrons so that they are not combined with photogenerated holes and thus minimizing energy loss in wasteful recombination. When no O_2_ purging was done, it produced only a trace amount of S_2_O_8_^2−^. It was further confirmed by analyzing the ORR in H_2_SO_4_ by making a thin film of catalysts over an FTO substrate, and Pt has shown the highest current density of oxygen reduction compared to various noble metals (Pd, Rh, Au, Ru) and bare FTO. The authors have proposed that photogenerated holes in the valence band of WO_3_ play an important role in the oxidation of HSO_4_^−^ to S_2_O_8_^2−^, whereas the role of Pd as co-catalyst has been suggested to participate in the reverse reaction of reducing back S_2_O_8_^2−^ to HSO_4_^−^. The effect of the electrolyte was examined by choosing H_2_SO_4_ and NaHSO_4_ at varying concentrations. The amount of S_2_O_8_^2−^ was gradually increased by increasing the electrolyte concentration, reaching a maximum at 1 M of H_2_SO_4_. On the basis of these findings, the mechanism of formation and decomposition of PDS over metal-loaded WO_3_ has been proposed. Authors have suggested that Pt-loaded WO_3_ has promoted O_2_ reduction to H_2_O_2,_ which is further reduced to H_2_O_2_. This has been found helpful for producing a higher concentration of PDS via an oxidation reaction catalyzed by valence band holes, whereas Pd-loaded WO_3_ has been suggested to participate in the decomposition of S_2_O_8_^2−^ to HSO_4_^−^. Overall, this study provides insight into the co-catalytic effect of noble metals on WO_3_ and reveals an important connection between ORR and S_2_O_8_^2−^ producing reactions. 

## 4. Advancements in Photoelectrochemical WO_3_ Photoanodes

Gu et al. have demonstrated an interface modulation approach in a microfluidic platform to boost the performance of WO_3_-based electrodes for PEC production of PDS [61]. Their designed system was composed of hierarchically structured WO_3_/TiO_2_ fabricated on porous carbon fiber as a substrate. The three-dimensional configuration of designed microfluidic channels has accelerated the PEC oxidation of HSO^4−^ anions and greatly enhanced the production yield and efficiency of PDS generation. The design of this work highlights the significance and potential of tailoring heterostructures for sustainable oxidant generation coupled with solar energy conversion. Providing a strong basis for engineering advanced nanostructures for solar to chemical conversions, maintaining high selectivity and efficiency.

The designed architecture has been favorable for boosting charge carrier separation and efficient mass transfer, which ultimately supported higher PEC performance for PDS generation. In this architecture, carbon fiber served as an affordable substrate for growing porous, three-dimensional WO_3_ nanostructures, whereas the TiO_2_ overlayer helped to suppress the recombination of photogenerated charge carriers. Adopting a photoelectrode instead of a dispersed catalyst could improve performance and make the catalyst easier to reuse. Therefore, instead of a dispersed slurry-based catalyst, photoelectrode fabrication can be considered as a preferred strategy for solar-assisted production of PDS. Mass transfer limitations in conventional photoelectrodes could hamper the higher yield of production formation. Microfluidics device design based on a 3D porous configuration, as adopted in this work, could promote efficient mass transfer phenomena leading to a higher rate of production formation. PL imaging techniques used to investigate the separation of charge carriers indicate that coupling TiO_2_ with WO_3_ has promoted interfacial charge transfer. A thin layer of TiO_2_ was coated on WO_3_, and the size of the TiO_2_ structure was observed to be around 50 nm. ESR analysis has confirmed the presence of oxygen vacancies in m-H-WO_3_ and m-H-WO_3_/TiO_2_ structures by the appearance of a signal corresponding to g = 2.003.

The electrode fabrication process was composed of seed-layer-mediated growth of the WO_3_ layer on carbon cloth. For that purpose, the substrate was dipped in a tungsten precursor solution and annealed at 400 °C. The process was repeated three times and then followed by hydrothermal growth of the WO_3_ layer on the seed-coated carbon fiber substrate. The hydrothermal process was repeated twice to grow a thick WO_3_ layer, followed by annealing at 500 °C for 2 h. Oxygen vacancies were introduced to the WO_3_ structure through reduction annealing at moderately higher temperature (350 °C) in the presence of a mixture 10% H_2_ in Ar. The prepared electrode was referred to as m-H-WO_3_. For the comparison, pl-H-WO_3_ was also prepared in a similar way by replacing the carbon cloth substrate with FTO. A TiO_2_ coating over m-H-WO_3_ was achieved using a wet chemical method by adopting ammonium hexafluorotitanate solution, followed by reductive annealing 10% H_2_ in Ar for creating oxygen vacancies. It was observed that the average diameter of carbon fiber was around 8 µm, whereas 2 µm thick WO_3_ nanoplates were grown on it. The TiO_2_ coating over WO_3_ plates was confirmed by elemental mapping.

The presence of oxygen vacancies was further confirmed by the appearance of two peaks at 34.8 eV and 36.6 eV in XPS analysis, assigned to 4f_7/2_ and 4f_5/2_ of W^5+^. JV characterization in the presence of Na_2_SO_4_ electrolyte has confirmed that m-H-WO_3_/TiO_2_ exhibits a higher current density than m-WO_3_/TiO_2_ and bare m-WO_3_. It shows that oxygen vacancies have played an important role in producing higher current density. The m-H-WO_3_/TiO_2_ has shown significant improvement in photocurrent density at 1.23 V vs. RHE compared to Pl-H-WO_3_ electrodes. These improvements in charge carrier separation and higher photocurrent generation have been reflected in a corresponding increase in the efficiency of PDS generation. After 120 min of PEC reaction, m-H-WO_3_/TiO_2_ has produced nearly 4 mM of S_2_O_8_^2−^ which is significantly higher than that observed for pl-H-WO_3_, being only 2.4 mM. It confirms the unique advantages of microfluidic design having a defective heterostructure for effective PDS production. 

**Figure 5:**
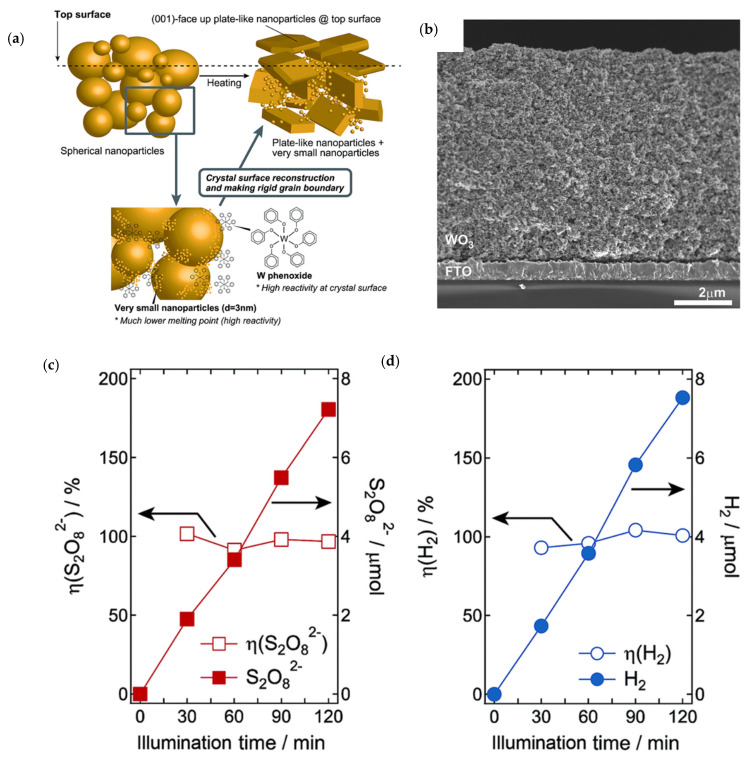
(**a**) Schematic illustration of crystal growth of WO_3_ by NPS hybrid dispersion ink, (**b**) cross-sectional view of FESEM for photoanode WO_3_, (**c**) time dependence of Faraday efficiency and generated amounts of (**a**) S_2_O_8_^2−^ (anode) and (**d**) H_2_ (cathode). The reaction was carried out under 1 Sun simulated sunlight illumination from the back of the W3 photoanode with a steady photocurrent at 0.2 mA adopted from reference [62].

Nakajima et al. have designed a WO_3_ nano sponge-based photoanode for PEC PDS production coupled with H_2_ production at the cathode over a Pt counter electrode [62]. The aim of this study was to improve the photocurrent density of the WO_3_ photoanode by tailoring inter-particle connections to enhance PDS production. The WO_3_ photoanode was fabricated using a nanoparticle/solution hybrid dispersion–deposition strategy as shown in Figure 5a. For that purpose, an ink was prepared in which WO_3_ was mixed with tungsten phenoxide in the presence of PEG. Spin coating and heating cycles were repeated to fabricate a thick WO_3_ film, having a thickness of several micrometers, as shown in Figure 5b. Nanopores in the photoanode contributed to achieving an absorbed photon-to-current efficiency of nearly 95.4% at 410 nm. The S_2_O_8_^2−^ was produced with Faradaic efficiencies for PDS at the anode and H_2_ at the cathode near to 100%, reflecting highly selective catalysis as shown in Figure 5c,d. Moreover, the applied bias photon-to-current efficiency was observed as 2.45% at 1.26 VCE. This is higher than the previously reported 2.2% efficiency for WO_3_ electrodes. The highest photocurrent observed for the optimized photoanode was about 3.04 mA/Cm^2^ at 1.05 V RHE.

Kusama et al. have performed DFT calculations to unfold the mechanism involving electrochemical oxidation of H_2_SO_4_ to PDS over WO_3_ and SnO_2_ anodes [66]. In all cases, proton-coupled electron transfer (PCET) was identified as the rate-determining step. Free energy changes have suggested SnO_2_-deposited WO_3_ is a more favorable anode for PDS production than SnO_2_ alone. Their experimental findings have further confirmed the higher Faradaic efficiency of WO_3_/FTO compared to FTO electrodes, reaching a maximum of 89% for 8 M H_2_SO_4_ at 10 mA for 100 s. Although this contribution does not use light irradiation, it provides useful insight about the preferred activity of PDS generation over WO_3_/FTO as compared to the bare FTO anode. Fuku et al. have fabricated porous WO_3_ electrodes for PEC-based PDS production coupled with H_2_ evolution [63]. The WO_3_ electrode applied in this work has been fabricated through spin coating of H_2_WO_4_ solution in the presence of polyethylene glycol. The optimized thickness of the WO_3_ electrode was around 2.7 um. Photocurrent generated from WO_3_ in H_2_SO_4_ electrolyte reflects superior response for back-side illumination as compared to front-side illumination. It is associated with better charge collection from back-side illumination.

The authors observed a correlation between H_2_SO_4_ concentration and the amount of PDS generation. A gradual increase in PDS generation was observed as the concentration of H_2_SO_4_ was increased to 1 M. Their fabricated electrode produced PDS with a Faradaic efficiency of nearly 100%, and ABPE of 2.2% was achieved at 1.13 V vs. CE. Although the developed WO_3_ electrode has also demonstrated oxidation of Ce^4+^ and IO_4_^−^, its efficiency remains lower compared to the PDS production process. Furthermore, it was integrated with dye-sensitized solar cells (DSSCs) to establish a bias-less PDS production process. Tandem design was achieved by connecting 2 DSSCs in series with WO_3_ as the photoanode and Pt as the cathode to produce PDS and H_2_, respectively. This tandem design has shown a highly stable photocurrent response over 1 h in the presence of H_2_SO_4_ electrolyte. Using this approach, 5.2% solar conversion efficiency was achieved by exploiting the absorption of longer wavelengths of radiation in the IR range. This work demonstrates an interesting concept of approaching tandem design for solar-assisted synthesis of PDS without applying external bias. It can be potentially extended to the synthesis of value-added chemical oxidants. 

Petruleviciene et al. achieved PEC-based generation of PDS over WO_3_ prepared by the solution deposition technique using different alcohols [64]. The thin films were annealed at different temperatures to monitor the effect of synthesis conditions on morphology and PEC performance. PEC performance was evaluated by measuring PDS production using H_2_SO_4_ solution. Alcohols (methanol, ethanol, isopropanol, and butanol) adopted in this synthesis acted as reductants for the synthesis of WO_3_. Two different heating conditions (400 °C and 500 °C) were used to anneal WO_3_ films. For the purpose of PEC characterization, a 0.5 M H_2_SO_4_ solution was used. PEC experiments were adopted using WO_3_ as the photoanode and Pt as the dark cathode to produce PDS and H_2_, respectively. WO_3_ prepared by annealing at a higher temperature of 500 °C has shown more enhanced crystallinity, whereas the sample annealed at 400 °C has shown inferior crystallinity as observed in the form of broad peaks in XRD patterns. The PEC response of WO_3_ electrodes annealed at 400 °C and 500 °C has revealed that those annealed at 400 °C exhibit better performance with earlier saturation of photocurrent density. The authors attribute this behavior to the efficient generation and separation of photogenerated charge carriers. Among various alcohols used as reductants, isopropanol has revealed the best photocurrent response. It was observed that the WO_3_ photoanode prepared in the presence of different alcohol reductants showed a decline in the efficiency for PDS generation upon prolonged irradiation, except for the photoanode prepared with IsoPrOH. Such samples have shown a rather improvement in Faradaic efficiency from 53% to 61%, while other samples have shown a significant decline. This work highlights the significant influence of electrode preparation parameters on the morphology, crystallinity, and stability of the photoanode, which ultimately influences the Faradaic efficiency of WO_3_ toward PEC-based PDS generation.

It is notable that the Faradaic efficiency of WO_3_-based electrodes toward PEC-based PDS production has been reported nearly to 100% in a few studies. It means that the bottleneck to improve the PDS production over WO_3_-based electrodes is to overcome the challenges in terms of photocurrent generation. If a higher amount of photocurrent generation is achieved, this could lead to an enhanced rate of PDS production. Although in the literature, several studies have been reported highlighting the effective modification of WO_3_-based photoanode to improve the photocurrent generation, there are still challenges to overcome the limitations of charge carrier recombination. Due to such factors, the photocurrent generation over WO_3_ remains low as compared to theoretically predicted values for WO_3_. In order to overcome such issues, several strategies have been explored to prevent the recombination of charge carriers to boost the PEC activity. Ahn et al., in an interesting work, have explored the role of the interfacial layer between substrate and WO_3_ photoanodes to prevent the unwanted recombination for improving the rate of PDS production [65]. Their study has concluded that a mesoporous blocking layer is more effective in preventing recombination when compared to a random particle-based layer. They have achieved an optimized photocurrent density of 2.6 mA cm^2^ at 1.23 V vs. RHE, resulting in PEC-based PDS production with a Faradaic efficiency reaching near to 95%. The interface between conducting substrates such as fluorine-doped tin oxide (FTO) and WO_3_ could play an important role in charge collection efficiency. Preventing the recombination at this interface can improve the availability of charges to effectively perform photo-driven redox reactions. The authors have investigated the effect of the morphology of WO_3_-based blocking layers on improving the PEC activity. For that purpose, a WO_3_-based blocking layer was introduced over the FTO substrate in two different forms: a randomly structured vs. a mesoporous WO_3_-based blocking layer. The random WO_3_ blocking layer was introduced by adopting the sol–gel method and introducing the precursor solution via spin coating. The mesoporous blocking layer has been introduced by the same approach, except adding poly(vinyl chloride)-g-poly(oxyethy lene methacrylate), as a grafting polymer in the solution. After spin coating, both blocking layers were heated at 500 °C for 30 min, followed by the growth of WO_3_ nanoflakes over them. PEC performance of fabricated electrodes has been investigated in the presence of 1 M H_2_SO_4_ while adding 0.5 M of H_2_O_2_ as a scavenger in it. There were clear morphological differences in the blocking layers prepared by both approaches. The random layer, owing to a bigger particle size and agglomeration, has exhibited voids in the film, whereas the mesoporous blocking layer has shown the continuity of the blocking layer. This has resulted in a clear difference in corresponding PEC activities. The variation in photocurrent density based on the thickness of the blocking layers highlighted that a moderately thin mesoporous layer has shown optimum performance during the PEC test. Corresponding IPCE results have further confirmed that mesoporous interlayers have superior performance as compared to random blocking layers. EIS analysis has confirmed that the random blocking layer has shown higher charge transfer resistance as compared to the mesoporous blocking layer. The WO_3_-based photoanode fabricated by introducing the mesoporous blocking layer has reflected the Faradaic efficiency of about 95%. This work highlights the significance of interface tuning to promote the PEC oxidation of H_2_SO_4_ for the efficient production of PDS as a valuable oxidant. 

Mi et al., in a pioneering work, investigated the suitability of WO_3_ photoanodes for oxidizing HSO_4_^−^ in the presence of aqueous and non-aqueous electrolytes, examining their influence on PEC oxidation of anions [67]. Various electrolytes investigated in this study include water, acetonitrile, and propylene carbonate. Besides oxidation of HSO_4_^−^, oxidation of other anions including CH_3_SO_3_^−^, HSO_4_^−^ and ClO_4_^−^. Efforts dedicated by authors to investigate the oxidation of anions in water and non-aqueous electrolytes provide useful insight into the PEC oxidation of the WO_3_ photoanode. WO_3_ photoanode was developed by electrodeposition on either FTO or “W” as a substrate. On account of exploring the photoelectrochemistry of the WO_3_ photoanode, various characterizations, including voltammogram, internal quantum yield, and electrochemical impedance analysis, have been performed. PEC oxidation of various anions, including HSO_4_^−^, in various solvents was examined. For the case of aqueous phase, 1.0 M of H_2_SO_4_ was used, whereas in the case of a protic solvent, 0.5 M of tetra(n-butyl) ammonium salt, such as TBAHSO_4_, has been adopted. The order of increasing internal quantum yield for HSO_4_^−^ oxidation over WO_3_/FTO in the presence of various electrolytes has been observed as follows: PC, 0.87 > ACN, 0.63 > water, 0.55. Mott–Schottky analysis has confirmed that electrolytes affect the flat-band potential of WO_3_/FTO anode, which has an influence on photocurrent onset. This study highlights the influence of various electrolytes of aqueous and non-aqueous compositions on the PEC oxidation of bisulfate anions.

**Figure 6:**
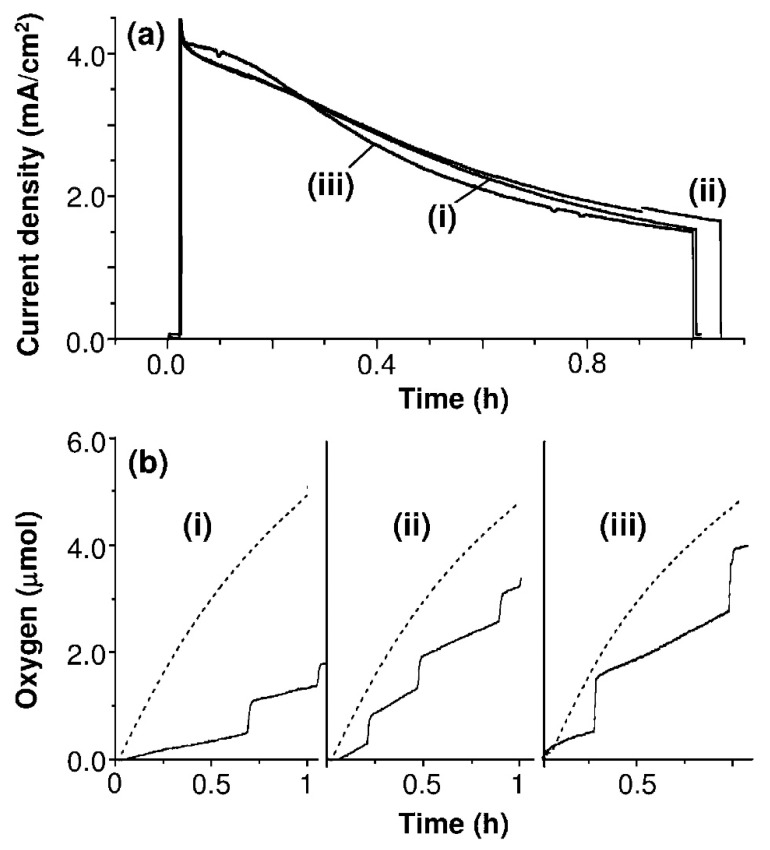
(**a**) Photocurrent and (**b**) oxygen measurements of a WO_3_ photoanode obtained at 0.9 V vs. Ag/AgCl in 0.1 M Na_2_SO_4_ solution with pH adjusted to (i) 1, (ii) 3, and (iii) 5 under 400 mW/cm^2^ illumination with an AM 1.5 G filter. In (**b**), actual amounts of O_2_ produced (−) are compared to expected amounts of O_2_ (- - -) calculated from photocurrent assuming 100% photocurrent to O_2_ conversion efficiency adopted from reference [35].

Hill et al. have also investigated the oxidation of sulfate anions (SO_4_^2−^) instead of HSO_4_^−^ to oxidize it into PDS over WO_3_ photoanode under different pH (1, 3 and 5) [35] on account of exploring long term stability, they have found that photocurrent was gradually decreased upon prolong illumination under all pH conditions such as 1, 3 and 5 as shown in Figure 6a. They further investigated the competing reaction of water oxidation to sulfate anion oxidation over the photoanode. Figure 6b shows the photocurrent density and O_2_ evolution rate vs. the theoretically expected, assuming 100% FE. The effect of various pH levels was reflected in the photocurrent to oxygen conversion efficiency, which was observed as 35%, 63%, and 88% for pH 1, 3, and 5, respectively. It indicates that when pH was higher, such as 5, it was more favorable for O_2_ evolution from water oxidation, while a more acidic pH, such as 1, was favorable for the formation of peroxo formation and sulfate oxidation to persulfate. This study confirms that sulfate oxidation to persulfate can be greatly affected by electrolyte pH; a more acidic pH could be more favorable for the sulfate oxidation reaction, suppressing water oxidation as a competing reaction. 

## 5. Challenges and Outlook

Although various strategies have been developed to enhance the capability of WO_3_ for light-induced PDS generation, there is ample room for further advances in this research direction. The critical challenges that remain unsolved are as follows: (i) production of a much lower amount and diluted concentration of PDS during the PEC oxidation process. (ii) The photocurrent generation is low, which limits the extent to which the application of PEC for large-scale production of PDS. (iii) Applying a dispersed or slurry type of photocatalyst to produce PDS under illumination has challenges in recovering the catalyst for reuse. (iv) Most of the work dedicated to PEC-based PDS production is limited to WO_3_, whereas the application of earth-abundant photoanodes remains less explored. (v) Exploiting suitable co-catalysts for selectively promoting PDS generation is lacking. (vi) There is a critical challenge of maintaining the stability of photoanodes during long-term applications, considering the corrosive nature of the electrolyte. To address the existing challenges, the following future research directions are suggested as follows: (i) In order to avoid the challenge of the lower production rate of PDS, the achievement of higher photocurrent generation over photoanodes should be focused on. This can be achieved by minimizing the recombination of photoinduced charge carriers. At present, photocurrent generation over WO_3_ photoanodes is lower than theoretically predicted values. Rationalized modifications of electrodes, such as heterostructure engineering and dopant introduction, can facilitate extended charge carrier separation. (ii) Use of metal-based co-catalysts should be extended to bimetallic configuration for tuning the work function and regulating charge transfer dynamics. It can effectively enhance charge carrier separation. (iii) Attention should be paid to passivating the surface of oxide photoanodes by coating the protective layers to avoid the problem of corrosion. (iv) Development of novel electrode materials that can sustain corrosive acidic electrolytes should be explored for diversifying PEC anodes for PDS generation. (v) Modification of photoanodes should be achieved to minimize the amount of overpotential needed to drive PDS production. (vi) The selectivity of PDS production should be tailored by reducing the occurrence of competing side reactions of OER at the photoanodes. (vii) Diverse strategies should be adopted to tailor the PEC response of photoanodes, including crystal facet engineering, preferred crystallographic orientation, defect engineering, tailoring oxygen vacancies and fabrication of bilayer and hierarchical three-dimensional nanostructures. (viii) Future research should focus on developing a paired PEC reaction with an appropriate reaction at the cathode and approaching an external bias-free PEC design that can solely rely on solar energy. It reduces the energy input needed to drive PDS production. (ix) To get maximum advantage of solar radiation as an abundant and free resource, such a photoanode should be designed that can absorb panchromatic radiation. It can extend the light absorption and harvesting capabilities. (x) DFT calculations should be performed for a deeper understanding of the reaction mechanism and unfolding the role of catalytic composition on boosting the selectivity and activity. We believe that investigating the above-suggested research direction can bring significant developments in developing advanced nanoscale architecture for light-assisted PDS production in an efficient manner.

## Figures and Tables

**Table 1:** Summary of PDS production over WO_3_-based photocatalysts.

Catalyst	Approach	Electrolyte	PDS Production Rate	Photocurrent	FE	ABPE	QE	Reference
Au@Pt/WO_3_	Photochemical	1.0 M H_2_SO_4_	36 μmol (3 h)	-	-	-	5.2% 420 nm	[59]
WO_3_/Pt	Photochemical	1.0 M H_2_SO_4_	33.9 μmol (3 h)	-	-	-	3.8% 420 nm	[60]
m-H-WO_3_/TiO_2_	PEC	0.5 M H_2_SO_4_ 0.5 M Na_2_SO_4_ (pH 6.0)	4 mM (2 h) 1.2 V vs. RHE	0.8 mA cm^−2^ at 1.23 V vs. RHE,	99% (20 min)	-	-	[61]
WO_3_	PEC	1.0 M H_2_SO_4_	Approx. ^a^ 7 μmol (2 h)	3.04 mA cm^−2^ at 1.50*V*_RHE_	Approx. ^a^ 100%	2.45% at 1.26*V*_CE_	-	[62]
WO_3_	PEC	1.0 M H_2_SO_4_	Approx. ^a^ 38 μmol (2 h)	Approx. ^a^ 2.7 at 1.5 V RHE	Approx. ^a^ 100%	2.2% at 1.13 V vs. CE	-	[63]
WO_3_	PEC	0.5 H_2_SO_4_	-	-	84% at CV 1.8 V	-	-	[64]
WO_3_	PEC	1.0 m H_2_SO_4_	Approx. ^a^ 36 μmol (2 h)	2.6 mAcm^−2^ at 1.23 V vs. RHE	95% at 1 mAcm^−2^	-	-	[65]

Approx. ^a^: Values are approximated from the Figures from corresponding references.

## Data Availability

No new data were created or analyzed in this study. Data sharing is not applicable to this article.
